# Myrrh protects against IL-13-induced epithelial barrier breakdown in HT-29/B6 cells

**DOI:** 10.3389/fphar.2023.1301800

**Published:** 2023-11-17

**Authors:** Helena Hader, Nina A. Hering, Jörg-Dieter Schulzke, Roland Bücker, Rita Rosenthal

**Affiliations:** ^1^ Department of Gastroenterology, Rheumatology and Infectious Diseases, Clinical Physiology/Nutritional Medicine, Charité—Universitätsmedizin Berlin, Berlin, Germany; ^2^ Department of General and Visceral Surgery, Charité—Universitätsmedizin Berlin, Berlin, Germany

**Keywords:** Myrrh, tight junction, barrier function, claudin, tricellulin, apoptosis, STAT pathway, inflammatory bowel disease

## Abstract

The oleoresin myrrh has been used for centuries as an anti-inflammatory remedy for a variety of diseases and is said to have a protective effect on the intestinal epithelium. An intact epithelial barrier function is the prerequisite for a healthy gut. Inflammatory and infectious diseases of the intestine, in particular, lead to barrier impairment resulting in leak-flux diarrhea and mucosal immune responses. Therefore, the aim of the present study was to investigate the protective effect of myrrh in an experimental inflammatory situation, namely, under the influence of IL-13, one of the key cytokines in ulcerative colitis. We used human intestinal epithelial HT-29/B6 cell monolayers for functional and molecular assessment of the epithelial barrier under IL-13 and myrrh treatment. IL-13 induced a loss in barrier function that was fully restored with myrrh treatment, as shown by transepithelial electrical resistance measurements. The molecular correlate of the IL-13-mediated barrier dysfunction could be assigned to an upregulation of the channel-forming tight junction (TJ) protein claudin-2 and to a subcellular redistribution of the TJ protein tricellulin, loosening the sealing of tricellular TJs. Moreover, IL-13 exposure leads to an increase in the number of apoptotic cells, contributing to the leak pathway of barrier dysfunction. Myrrh protected against changes in TJ deregulation and decreased the elevated apoptotic ratio under IL-13. The protective effects are mediated through the inhibition of the STAT3 and STAT6 pathway. In conclusion, our results demonstrate that myrrh exhibits antagonizing effects against IL-13-induced barrier impairment in a human intestinal cell model. These data suggest the use of myrrh as a promising option in the treatment of inflammatory bowel disease.

## Introduction

An impaired intestinal barrier function is a key mechanism in inflammatory bowel disease (IBD), a term that comprises Crohn’s disease (CD) and ulcerative colitis (UC). Currently, IBD patients are commonly treated with immunosuppressive medication, causing numerous side effects (Kucharzik et al., 2019). For this reason and as the incidence of IBDs is globally increasing (Arms-Williams et al., 2022), it is crucial to examine other target substances of herbal origin, such as myrrh, as potential treatment options.

While the exact etiology of CD and UC remains unresolved, it was shown that the increased expression of the proinflammatory cytokines tumor necrosis factor alpha (TNFα) and interferon gamma (IFNγ), as well as interleukins (IL), such as IL-2 and IL-13, cause an intestinal epithelial barrier dysfunction, which in turn facilitates the entry of antigenic macromolecules, toxins, and bacteria, promoting leak-flux diarrhea and the leaky gut phenomenon (Hering and Schulzke, 2009; Turner, 2009). Especially IL-13 has been identified as an important effector cytokine in UC (Heller et al., 2005). Predominantly known for the effect of IL-13 in immune-mediated processes and allergic inflammation, it has been shown that patients with IBD express higher amounts of IL-13 in their lamina propria cells than found in the gut of healthy individuals (Fuss et al., 2004). Via the receptors IL-13Rα1 and IL-4Rα, IL-13 impairs epithelial barrier function by affecting epithelial apoptosis, tight junctions (TJs), and restitution velocity in UC (Heller et al., 2005).

The TJ connects intestinal epithelial cells at their most apical point of the lateral membrane, forming a single continuous layer of enterocytes (Chiba et al., 2008). TJs ensure barrier integrity, control paracellular transport processes and determine the polarization of the cell (Zihni et al., 2016). TJs are composed of transmembrane proteins belonging to different families: claudins, tight junction-associated MARVEL proteins (TAMPs) and junctional adhesion molecules (JAMs). Especially claudins and TAMPs play an integral role for barrier function and encompass both sealing and selective channel-forming properties (Günzel and Fromm, 2012). In addition, intracellular scaffold proteins, namely, ZO-1 and ZO-2 (Zonula occludens proteins-1 and -2) link the transmembrane TJ proteins to other TJ-associated proteins and to the intracellular actin cytoskeleton (Chiba et al., 2008).

By regulating the expression and localization of TJ proteins, certain phytochemicals, such as berberine (Amasheh et al., 2010), quercetin (Amasheh et al., 2008), shogaol (Luettig et al., 2016), or punicalagin metabolites (Hering et al., 2021) are already known to exert barrier-stabilizing effects. For instance, berberine protects against TNFα-mediated barrier impairment and TJ dysregulation (Amasheh et al., 2010; Luettig et al., 2016). Quercetin enhances barrier function via upregulation of ZO-1 and -2, claudin-1 and -4 (Amasheh et al., 2008; Suzuki and Hara, 2009). Moreover, *in vivo* as well as *in vitro* experiments have shown that curcumin and resveratrol have barrier-protective properties during a *Campylobacter jejuni* infection (Lobo de Sá et al., 2019; Lobo de Sá et al., 2021).

A further ethnopharmaceutical is myrrh, a term referring to the dried oleo-gum resin obtained from the bark of *Commiphora myrrha*, a tree native to North Africa and the Arabian Peninsula. The compound consists of 30%–60% water-soluble gums, 25%–40% alcohol-soluble resin and 3%–8% essential oils (Kuck, 2021). It has been used for religious, cosmetic and medical purposes since antiquity (Shen et al., 2012). First remedies containing myrrh were described by Dioskurides in his *Materia medica* (Dioskurides, 2005). The medicinal herb was used for chronic cough, abdominal pain and diarrhea. In modern times, myrrh is mainly used in mouthwash solutions and for the treatment of small skin lesions (Shen et al., 2012). Currently, there are two preparations for the treatment of other diseases available on the global market: Mirazid, an antischistosomal medication that has been introduced in 2001 to the Egyptian market (ClinicalTrials.gov, 2015), and Myrrhinil Intest, a combined preparation of myrrh, chamomile and coffee charcoal, which has been found to be non-inferior against mesalazin, the current gold standard remission maintenance medication in UC (Langhorst et al., 2013). The Myrrhinil Intest drug was effective in the treatment of acute diarrhea caused by irritable bowel syndrome (IBS) and in maintenance of remission in UC. In experiments conducted *in vitro*, myrrh exhibits barrier-stabilizing and spasmolytic effects in intestinal cells (Weber et al., 2020). The anti-inflammatory effect of myrrh was shown for various pro-inflammatory cytokines, chemokines and prostaglandins in intestinal and immune cells (Manjula et al., 2006). Myrrh exerts barrier-stabilizing and -protective effects against TNFα in HT-29/B6 and Caco-2 intestinal epithelial cells (Rosenthal et al., 2017). Myrrh extract was able to reduce the production of CXCL13, IL-6, IL-8, PGE2, MCP-1, IL-10 and TNFα in macrophages (Vissiennon et al., 2017). Furthermore, previous studies showed the protective effect of myrrh in murine models of DSS-, TNBS- and oxazolone-induced colitis (Cheon et al., 2006; Mencarelli et al., 2009). Therefore, the aim of this study was to decipher the molecular mechanisms and the involved signaling pathways of myrrh’s protective effect on epithelial barrier function and against the proinflammatory cytokine IL-13.

## Methods

### Cell culture and experimental setup

HT-29/B6 cells, a subclone of the human colon adenocarcinoma cell line HT-29 (Kreusel et al., 1991), were cultured in 25 cm^2^ culture flasks in RPMI 1640 medium (Thermo Fisher Scientific, Waltham, MA, United States) supplemented with 10% fetal calf serum (Thermo Fisher Scientific, Waltham, MA, United States) and 1% penicillin/streptomycin in a humidified atmosphere (95% air, 5% CO_2_) at 37°C. The cells were seeded on 3.0 μm filters (Merck Millipore, Billerica, MA, United States) and up to four filters were placed into one culture dish. Seven to eight days after seeding, the cell monolayers reached confluence (600–800 Ω cm^2^ transepithelial electrical resistance) and were used for experiments. The ethanolic extract of the herbal compound myrrh was provided by Prof. Jörg Heilmann, (Institut für Pharmazie, Lehrstuhl für Pharmazeutische Biologie, Universität Regensburg, Germany) and prepared as stock solutions of 500 mg/mL in 100% ethanol or 200 mg/mL in 100% dimethyl sulfoxide (DMSO). To mitigate the cytotoxic effects of ethanol and DMSO, the final utilized concentration of the solvents was below 0.1%. Prior to stimulation with IL-13, the cells underwent 2 h of preincubation with myrrh. 200 μg/mL myrrh was added to cell medium and dissolved in a sonification bath before application on the apical and basolateral side of the filter. Subsequently 10 ng/mL IL-13 (Peprotech, Hamburg, Germany) was added basolaterally. Likewise, the histone acetylation inhibitor SAHA exhibiting STAT3 and STAT6 inhibition was applied 2 h before IL-13 exposure (10 μM SAHA, Cat. 149647-78-9, Sigma Aldrich, Schnelldorf, Germany, Axon Medchem, Groningen, Netherlands; 10 µM SAHA is effective in blocking STAT6; Rosenthal et al., 2017). In experiments aiming at the signaling pathways cell monolayers were serum starved for 4 h, followed by a two-hour incubation period with myrrh. After incubation with IL-13 for 1 h, the cells were scraped and lysed for Western blot experiments. To detect apoptotic cells, levels of caspase-3/-7 activity were determined using the SensoLyte Homogeneous AFC Caspase-3/7 Assay kit according to manufacturer’s instructions (Biotrend Chemikalien GmbH, Cologne, Germany).

### Electrophysiological and flux measurements

The transepithelial electrical resistance (TER) of the monolayer was assessed under sterile conditions at 37°C. The measurements were mechanically standardized using a pair of chopstick electrodes (STX2, World Precision Instruments, Sarasota, FL, United States) with constant immersion depth. One electrode was placed on each side of the filter while in the culture dish and the electrical resistance was obtained using a volt-ohm meter (constructed in the Clinical Physiology, Charité - Universitätsmedizin Berlin) referred to an empty filter and the cell medium as blank. Data is presented in percentage of the initial resistance before treatment with the respective substances. Unidirectional flux measurements were performed in Ussing chambers under short-circuit conditions as described before (Günzel et al., 2009). Following equilibration, the paracellular flux marker fluorescein isothiocyanate (FITC)-dextran 4 kDa (FD4, TdB Consultancy, Uppsala, Sweden) was added apically (final concentration 200 μM). Samples were taken from the basolateral side every 20 min and analyzed by spectrophotometry (Tecan GmbH, Männedorf, Switzerland). FD4 permeability is presented as the quotient of flux and concentration difference.

### Western blotting

For protein expression analysis via Western blot the cells were rinsed two times with ice-cold phosphate buffered saline (PBS, Thermo Fisher Scientific, Waltham, MA, United States) and homogenized with whole cell lysis buffer (10 mM Tris, 150 mM NaCl, 0.5% Triton X-100% and 0.1% sodium dodecyl sulphate (SDS), one tablet Complete Protease Inhibitor Cocktail/10 mL lysis buffer (Roche, Basel, Switzerland)). For phosphoblots, buffer solution containing 20 mM Tris, 150 mM NaCl, 1 mM EDTA, 1 mM EGTA, 2.5 mM Na_4_P_2_O_7_, 1 mM Na_3_VO_4_, 1 mM PMSF, 1 mM β-glycerophosphate, 1% Triton X-100, 1 μg/mL leupeptin and protease inhibitors was used. Subsequently, the cells were carefully scraped from the filter. The lysate was incubated on ice for up to 60 min, followed by centrifugation for 15 min at 15,000 x g and 4°C. The supernatant was transferred into fresh vials. To determine the protein content the Pierce BCA kit was used according to manufacturer’s instructions (Thermo Fisher Scientific, Waltham, MA, United States). Proteins were resolved in polyacrylamide gels ranging from 8.5%–12.5% at 100 V and transferred to a nitrocellulose membrane (Perkin Elmer, Rodgau, Germany) after electrophoresis. Overnight, the membranes were incubated at 4°C with primary antibodies against TJ proteins, signaling pathway proteins as well as their phosphorylated forms (1:1,000, claudin-1 (Cat. 519000), claudin-2 (Cat. 325600), claudin-3 (Cat. 341700), claudin-4 (Cat. 329400), claudin-5 (Cat. 352500), claudin-7 (Cat. 349100), claudin-8 (Cat. 400700Z), tricellulin (Cat. 700191), occludin (Cat. 711500), Invitrogen, Carlsbad, CA, United States) or β-actin (1:10,000, Cat. AM4302, Invitrogen, Carlsbad, CA, United States). After rinsing with tris-buffered saline-Tween solution (TBST), incubation with secondary antibodies (goat anti-rabbit and goat anti-mouse in TBST with 1% milk powder) for 1 h followed (1:10,000 (Cat. 111-036-003 and Cat. 115-036-003), Jackson ImmunoResearch, Cambridge House, United Kingdom). Signal detection was conducted after incubation for 5 min in SuperSignal West Pico PLUS Stable Peroxide Solution (Thermo Fisher Scientific, Waltham, MA, United States), using Fusion FX7 imaging system. Image Studio Lite (version 5.2, 2015, LI-COR Biosciences Inc., Lincoln, NE, United States) was used for densitometric quantification. The TJ protein or phosphoprotein expression levels were normalized to β-actin or unphosphorylated form as loading control.

### Immunofluorescence staining

For immunofluorescence stainings, cells were washed with PBS and fixed for 10 min in 4% paraformaldehyde (PFA, Thermo Fisher Scientific, Waltham, MA, United States). After 3 washing steps with PBS, permeabilization with 0.5% Triton X-100 for 10 min and incubation for 1 h in blocking solution (containing 5% goat serum, 0.05% Triton X-100, and 1% bovine serum albumin (BSA)) followed. Primary antibodies against TJ proteins (claudin-2 (Cat. 325600, 1:100), tricellulin (Cat. 700191, 1:200), occludin (Cat. 711500, 1:100) and ZO-1 (Cat. MA3-39100-A647, 1:100), Invitrogen, Carlsbad, CA, United States) were added for incubation overnight. After rinsing with blocking solution, the cells were incubated with anti-mouse or anti-rabbit secondary antibodies conjugated to Alexa-Fluor 488 or 594 for 1 h (1:200) at 37°C (Cat. A11034 and Cat. A11032, Invitrogen, Carlsbad, CA, United States). Nuclei were counterstained with 4′,6-diamidino-2-phenylindole (DAPI, 1:1000, Cat. 10 236 276 001, Roche AG, Mannheim, Germany) for 10 min. Stainings using the TUNEL kit according to manufacturer’s instructions (*In situ* Cell Death Detection Kit, Roche AG, Mannheim, Germany) were used for apoptosis quantification. Cells were embedded in ProTaqs MountFluor (Biocyc, Luckenwalde, Germany) and visualized with confocal laser scanning microscopy (Zeiss 780, Zeiss, Jena, Germany).

### Statistics

Data are shown as arithmetic mean ± standard error of the mean (SEM). One-way-ANOVA and Bonferroni-Holm correction for multiple comparisons were used to perform statistical analysis. Analyses were carried out with GraphPad PRISM (version 9.0, 2020, GraphPad Software, Inc. San Diego, CA, United States). A *p*-value smaller than 0.05 was considered significant. *p* < 0.05 depicted as *, *p* < 0.01 as ** and *p* < 0.001 as ***.

## Results

### Myrrh restores barrier function after IL-13-induced impairment

**FIGURE 1:**
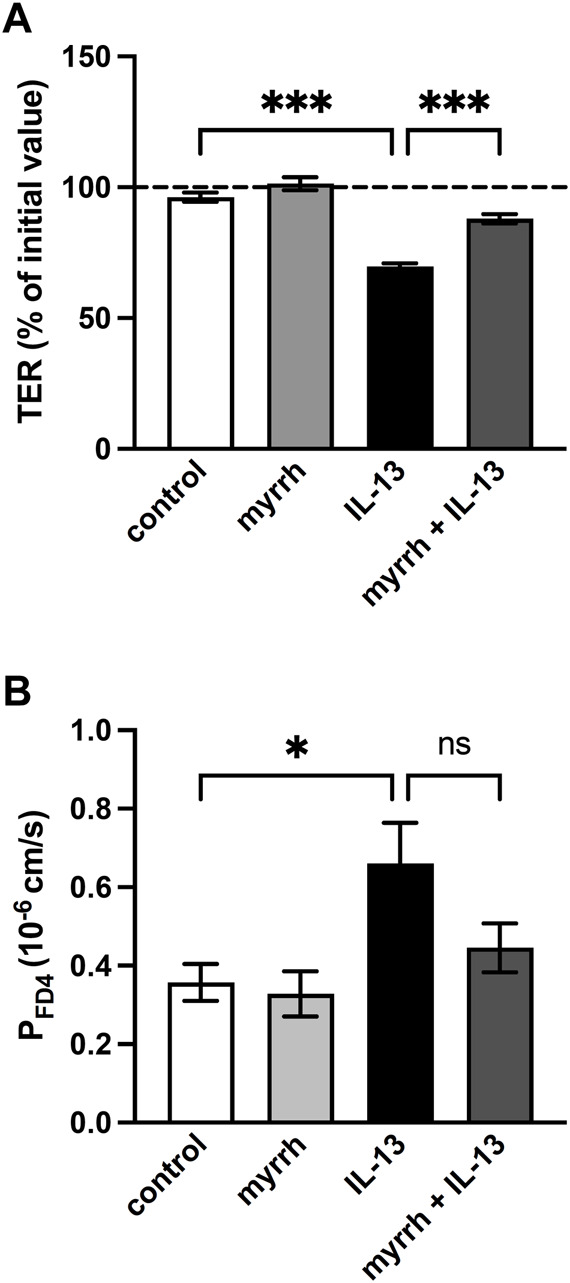
Effect of myrrh on the epithelial barrier defect caused by IL-13. HT-29/B6 cell monolayers were incubated for 2 h with 200 μg/mL myrrh, following basolateral incubation with 10 ng/mL IL-13. **(A)** Effect on transepithelial electrical resistance (TER). TER was measured 24 h after treatment. Control was set to 100%. Myrrh had an inhibitory effect against the IL-13-induced decrease in TER. *n* = 15–18. **(B)** Effect on FITC-dextran 4 kDa (FD4) permeability. Transepithelial flux was measured in Ussing chambers using the paracellular marker FD4. IL-13 caused an increase in permeability to FD4, which myrrh reduced, but this did not reach statistical significance. N = 8, ns = not significant, *p < 0.05, ***p < 0.001, one-way ANOVA with Bonferroni correction.

How the cytokine IL-13 causes intestinal barrier disruption via various mechanisms has previously been described (Heller et al., 2005). In order to investigate a potential protective effect of myrrh on IL-13-induced epithelial barrier dysfunction, the TER of HT-29/B6 monolayers was measured. Previous experiments have used a concentration of 200 μg/mL myrrh to influence TER, without cytotoxic side effects (Rosenthal et al., 2017). Measurements were performed over an incubation period of 24 h. Incubation with 200 μg/mL myrrh alone did not change the TER value significantly, although a slight increase of 5% could be observed compared to controls (myrrh: 101% ± 2%, control: 96% ± 2%, n = 15–18). Treatment with the proinflammatory cytokine IL-13 (10 ng/mL) caused a drop in TER of 30% after 24 h (70% ± 2%, n = 18, *p* < 0.001). Cells exposed to IL-13, but pre-incubated for 2 h with 200 μg/mL myrrh did show an attenuated decrease (88% ± 2%, n = 15, *p* < 0.001, Figure 1A). Considering the TER as a measure for the tightness of an epithelial layer, myrrh exhibits potential protective properties.

### Paracellular permeability under IL-13 and myrrh exposure

While the TER is a marker for the ion permeability of the epithelial barrier, we determined in the following step whether myrrh has protective properties concerning the paracellular permeability of larger molecules. In Ussing chambers, flux measurements of the paracellular marker FITC-dextran with a molecular mass of 4 kDa (FD4) were performed for calculation of the FD4 permeability. Compared to controls, IL-13 caused an increase in paracellular permeability for FD4 (0.66 ± 0.10·10^−6^ cm/s, n = 8, *p* = 0.027, Figure 1B). The preincubation with myrrh reduced the effect of IL-13 on the permeability increase but failed to reach statistical significance (0.45 ± 0.06·10^−6^ cm/s, n = 8, *p* = 0.168, Figure 1B).

### Effect of myrrh on IL-13-mediated changes in tight junction protein expression and localization

#### Myrrh inhibits claudin-2 upregulation induced by IL-13

**FIGURE 2:**
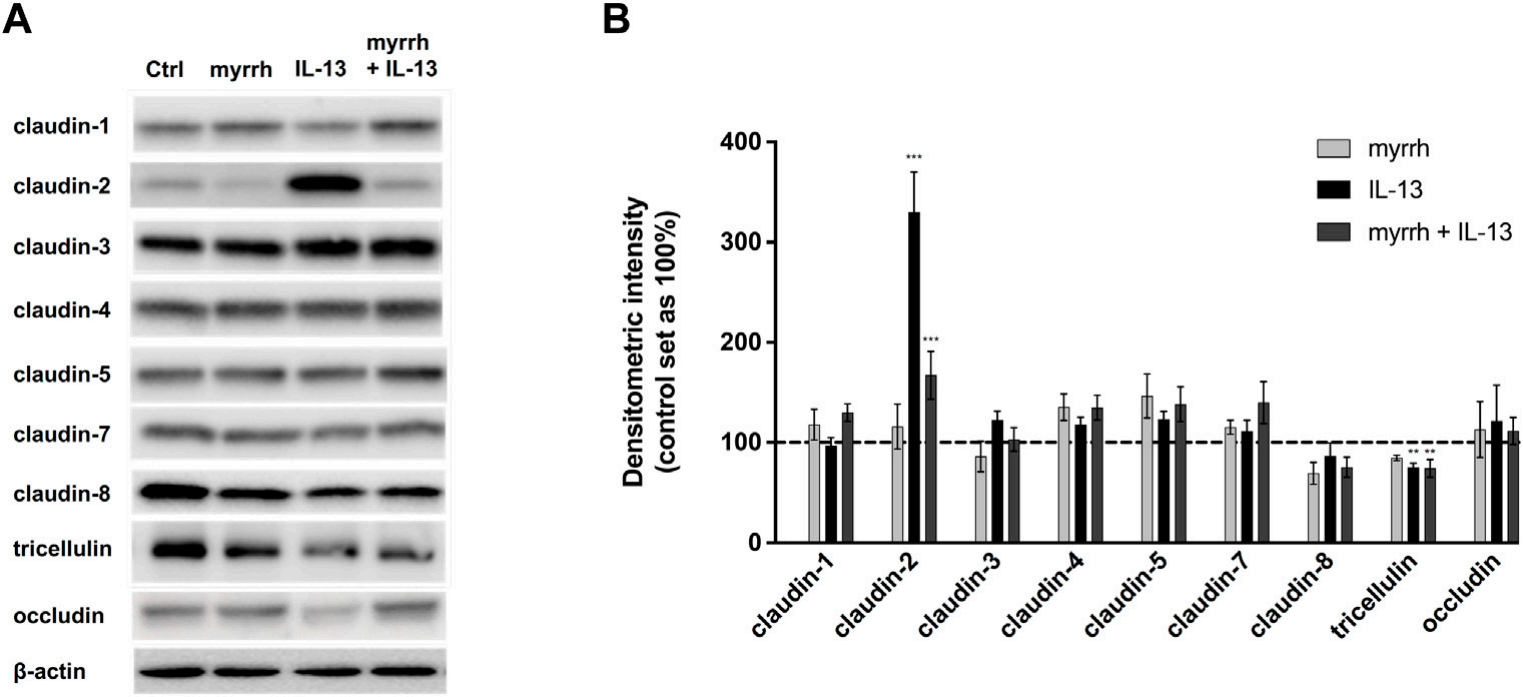
Effect of IL-13 or myrrh and the combination of both on tight junction protein expression in HT-29/B6 cells. **(A)** Representative Western blots of epithelial TJ proteins after myrrh and IL-13 incubation. Proteins were isolated following 24 h of treatment and quantified using densitometric analysis, normalized to β-actin **(B)**. Expression of TJ proteins was altered as a result of IL-13 and myrrh treatment. IL-13 caused an increase in claudin-2 that was inhibited by myrrh. A decrease in tricellulin under IL-13 treatment was observed, which did not change by myrrh preincubation. Other TJ proteins were not affected by myrrh or IL-13. N = 6–12, **p < 0.01, ***p < 0.001, one-way ANOVA with Bonferroni correction. Comparisons were made between IL-13-treated cells and untreated controls. Comparisons were also made between the group of IL-13 plus myrrh treatment versus the cells treated with IL-13 alone.

To investigate whether myrrh protects the epithelial barrier by influencing the composition of TJ proteins, Western blot analyses were performed (Figure 2). The expression of the claudins-1 to −8 as well as of tricellulin and occludin was quantified by densitometry. All claudins except the channel-forming claudin-2 remained unchanged after treatment with IL-13 or myrrh (Figure 2).

Densitometric analysis showed a three-fold increase in claudin-2 expression in cells incubated with IL-13 (compared to controls, n = 12, *p* < 0.0001). Myrrh alone does not induce a significant change in claudin-2 levels compared to controls (compared to controls, n = 12, *p* = 0.9792). The incubation with myrrh before challenge with IL-13 counter-regulates the IL-13 effect, leading to a strong decrease in claudin-2 expression compared to IL-13-treated cells (compared to controls, n = 12, *p* = 0.0006).

Furthermore, a decrease in tricellulin expression was observed in cells treated with IL-13 (Figure 2). IL-13 reduced the expression of tricellulin to 75% ± 4% compared to controls (n = 10, *p* = 0.0034). This decrease in expression was not attenuated by myrrh (74% ± 10%, n = 10, *p* = 0.5079).

#### Subcellular tight junction protein localization

**FIGURE 3:**
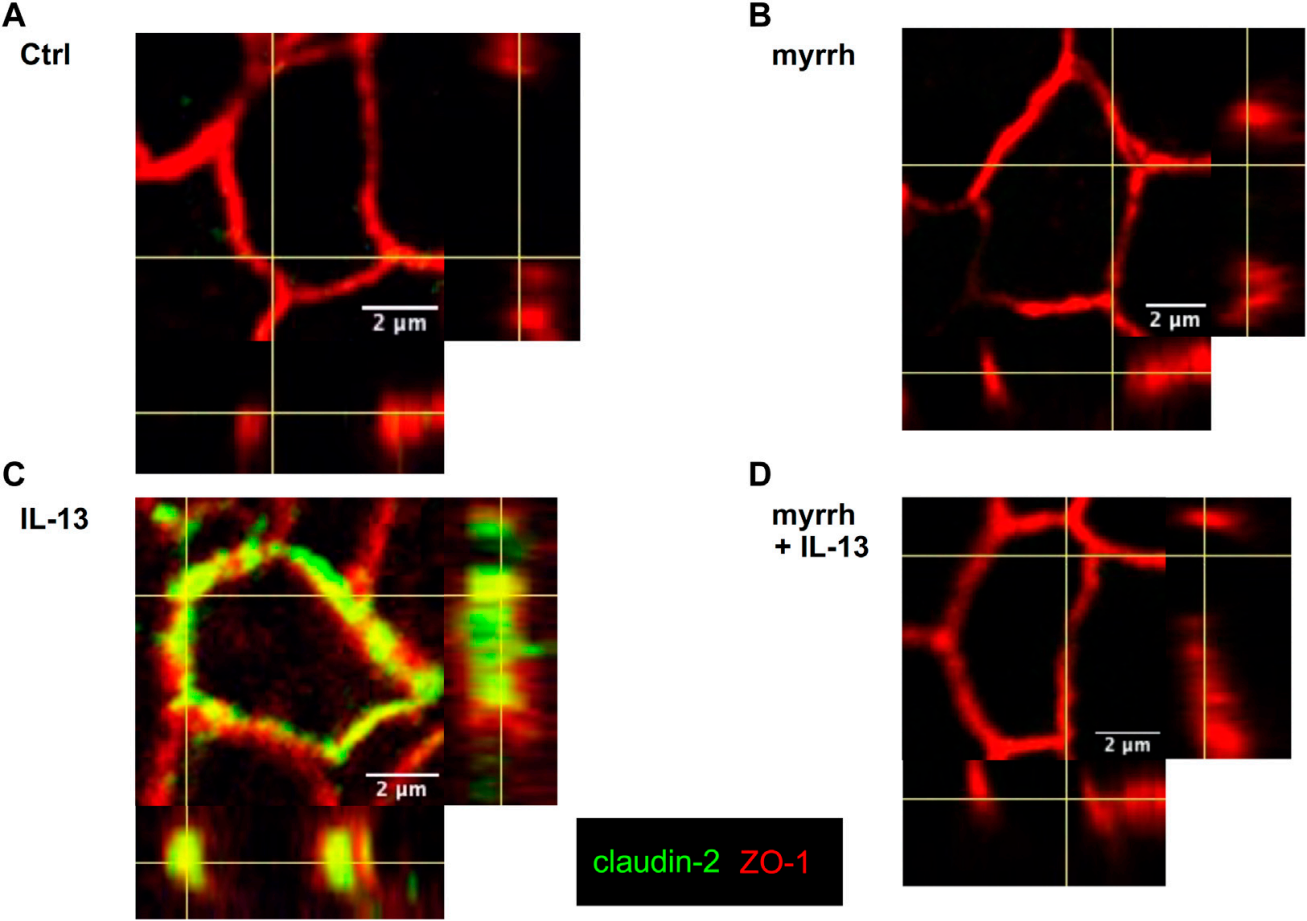
Claudin-2 distribution in HT-29/B6 cells after incubation with IL-13 and myrrh. Representative immunofluorescence stainings of claudin-2 (green) and Zonula occludens protein-1 (ZO-1, red), obtained in *Z*-stacks using confocal laser-scanning microscopy. **(A)** Control **(B)** myrrh **(C)** IL-13 **(D)** myrrh + IL-13. In control monolayers and myrrh-treated cells, claudin-2 was not detectable. IL-13 induced an upregulation and incorporation of claudin-2 into the membrane. Under treatment with myrrh, claudin-2 could not be detected.

In addition to the changes in TJ protein expression, seen in Western blots, an altered protein localization may also contribute to barrier defects. While Western blots are a quantitative measurement for protein expression, immunofluorescence staining provides information on protein localization and thus on TJ architecture. Thus, confocal laser-scanning microscopy was used to investigate TJ protein localization in presence of myrrh and IL-13. The TJ proteins claudin-1 to −8 were counterstained with ZO-1, tricellulin was stained together with occludin. There were no alterations found for claudin-1 and for claudin-3 to −8 (data not shown). Controls and cells treated only with myrrh did not show a visible amount of claudin-2 in their membranes as shown in Figures 3A, B. Corresponding to the Western blot results, cells incubated with IL-13 did show a higher incorporation of claudin-2 into the plasma membrane of the epithelial cells (Figure 3C). In cells preincubated with myrrh before undergoing treatment with IL-13, the claudin-2 signal was reduced and was comparable to the (non-visible) control level (Figure 3D).

**FIGURE 4:**
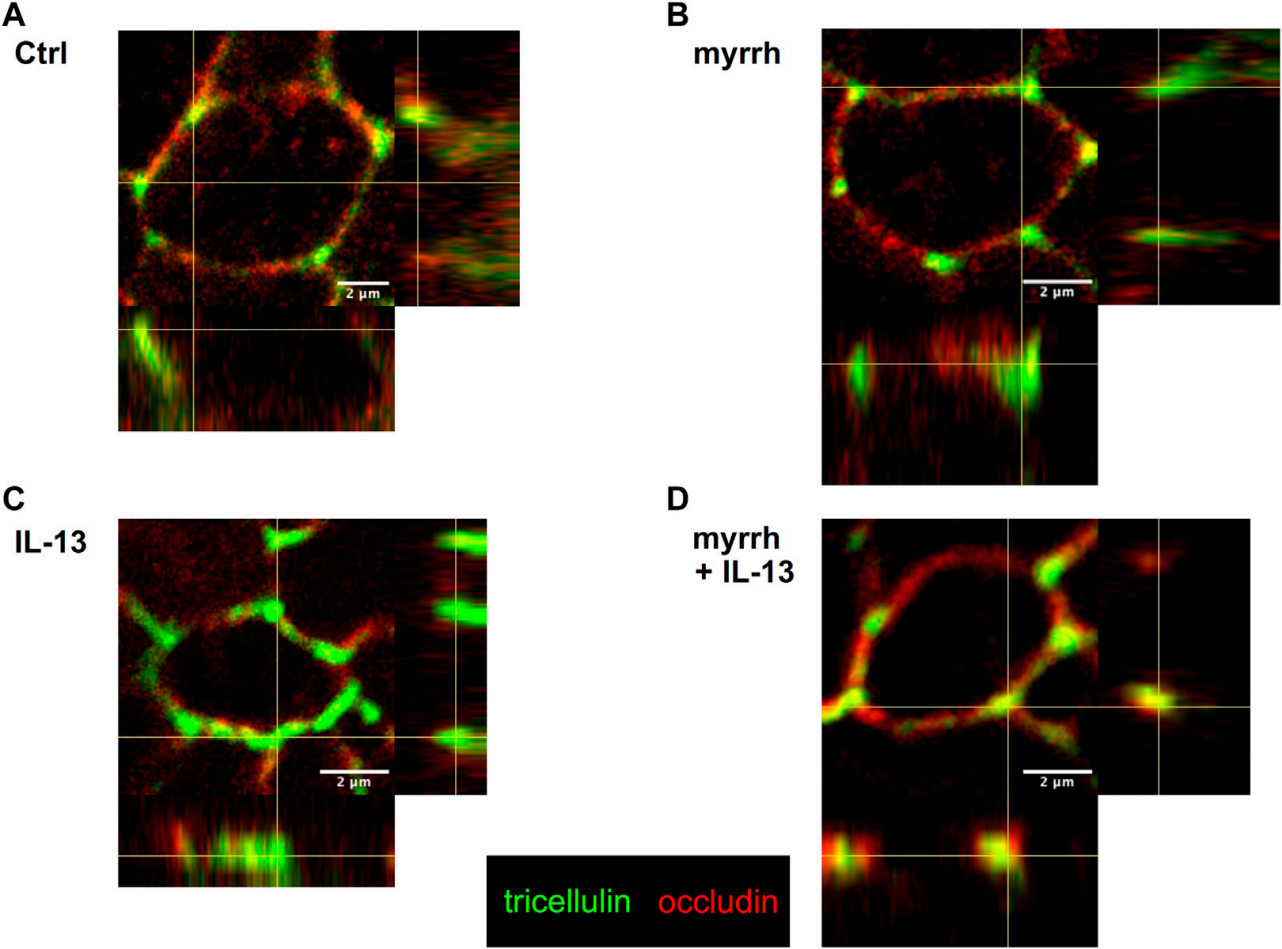
Tricellulin distribution after incubation with IL-13 and myrrh in HT-29/B6 cell monolayers. Representative immunofluorescence stainings of tricellulin (green) and occludin (red), obtained in *Z*-stacks using confocal laser-scanning microscopy. **(A)** Control, **(B)** myrrh, **(C)** IL-13 and **(D)** myrrh + IL-13. In controls and cells incubated with myrrh, tricellulin appears exclusively in the tricellular TJ and does not show any redistribution. IL-13 induces a shift of tricellulin into the bicellular plasma membrane, which is attenuated by myrrh.

Additionally, an altered distribution of tricellulin was observed. In controls and cells treated with myrrh, tricellulin appeared well-localized in the tricellular junction (Figures 4A, B). Under challenge with IL-13, tricellulin was found to be redistributed from tricellular TJ domain into the bicellular TJ domain, as seen in Figure 4C. Remarkably, the treatment with myrrh reversed this redistribution of tricellulin (Figure 4D).

### Protective effect of myrrh on epithelial apoptosis caused by IL-13

**FIGURE 5:**
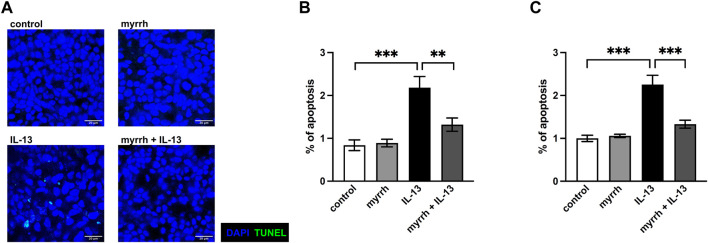
Inhibitory effect of myrrh on IL-13-induced epithelial apoptosis in HT-29/B6 cells. **(A)** Representative images of apoptotic cells, detected with TUNEL stainings. Control, myrrh, IL-13 and myrrh + IL-13. **(B)** Corresponding apoptotic cell rates to the TUNEL stainings. **(C)** Apoptosis was determined by caspase-3/-7 activity, caspase-3/-7 activity of control cells was set = 1. IL-13 induced a higher apoptotic rate. Myrrh incubation protects against the induction of apoptosis. N = 9–12 ***p < 0.001, one-way ANOVA with Bonferroni correction.

A relevant mechanism for a decrease in TER can be a higher apoptotic rate (Heller et al., 2008). In order to examine whether myrrh takes effect on the epithelial barrier by changing the apoptotic rate of HT-29/B6-monolayers, we performed TUNEL stainings for cell death counting. After 24 h of treatment, cells were stained with DAPI to detect cell nuclei and TUNEL reagent for cell death detection. The cell monolayers were visualized with confocal laser-scanning microscopy (Figure 5A). The apoptotic rate was determined by dividing the TUNEL-positive cells by total cell count. In IL-13-challenged monolayers, the apoptotic rate increased (Figure 5B). The apoptotic effect of IL-13 was inhibited by myrrh preincubation (Figure 5B). These observations were confirmed by measuring caspase-3 and -7 activity for apoptosis detection. Cells incubated with myrrh alone did not show a significantly lower or higher apoptotic rate compared to controls (Figure 5C, n = 9–12, *p* = 0.991). However, exposure to IL-13 induces a two-fold increase in apoptotic rate (Figure 5C, n = 12, *p* < 0.001). This higher apoptotic rate was decreased by preincubation with myrrh and restored the ratio to control levels (Figure 5C, n = 12, *p* < 0.001). Therefore, the barrier-protecting effect of myrrh is partly due to the prevention of apoptotic leaks induced by IL-13.

### Involved signaling pathways

**FIGURE 6:**
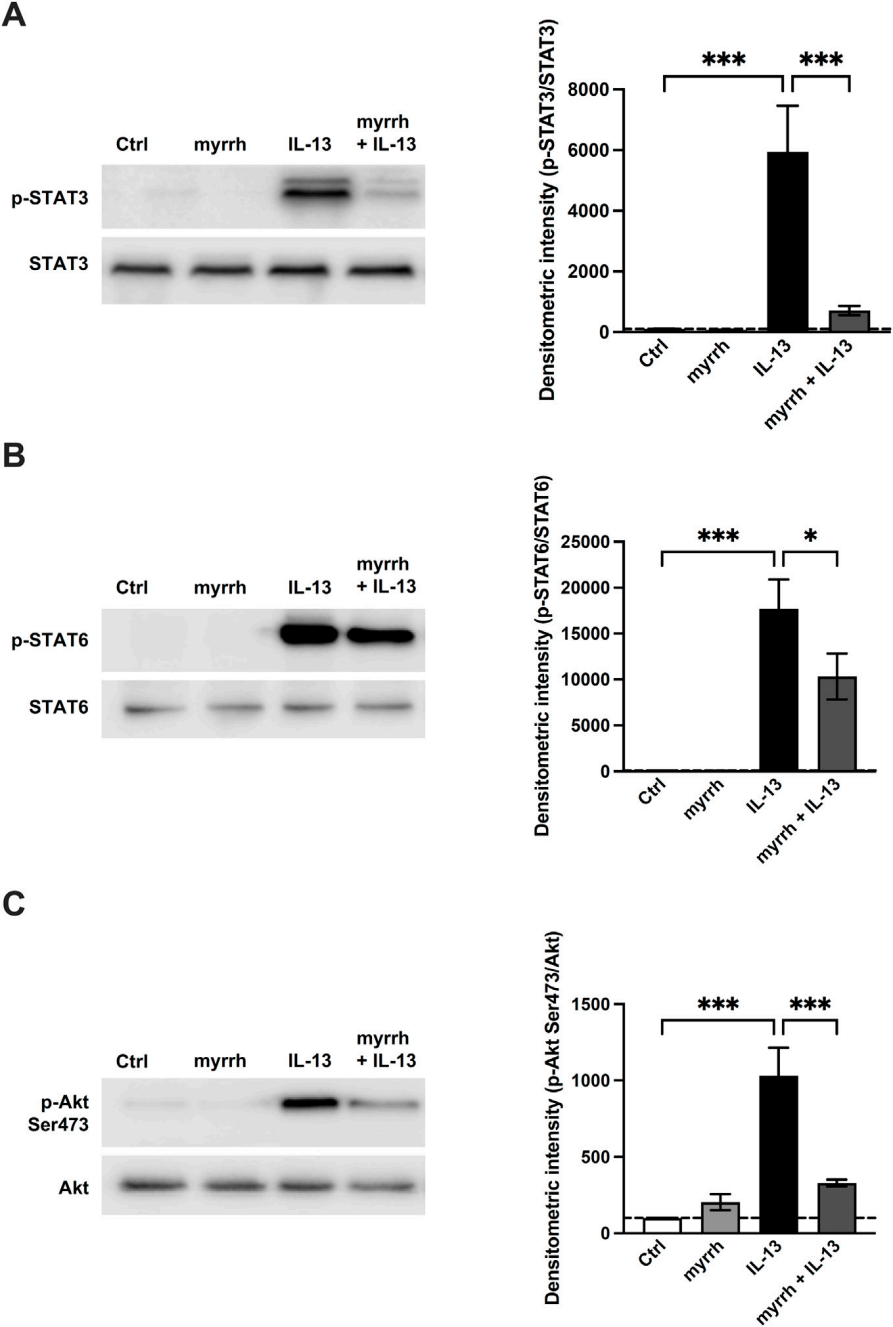
Effect of myrrh on IL-13-induced signaling pathways. Analysis of signaling pathways. Representative Western blots of phosphorylated proteins involved in **(A, B)** JAK/STAT, **(C)** PI3K and MAPK signaling, normalized to their total amount. Densitometric intensity of **(A)** p-STAT3, **(B)** p-STAT6 and **(C)** p-Akt Ser473 is increased in cells treated with IL-13. Myrrh attenuates this increase. *n* = 8–12, *p < 0.05, *p < 0.001, one-way ANOVA with Bonferroni correction.

To uncover the underlying signaling pathways involved in the barrier-stabilizing effect of myrrh, we analyzed the phosphorylation of signaling proteins by Western blots. Following incubation with myrrh for 2 h, HT-29/B6 cells were exposed to IL-13 for 1 h and lysed afterwards. Western blots for detection of the phosphorylation of JAK/STAT, STAT3, STAT6, Akt Ser473, Akt Thr308, p-38 and p-42/44 were carried out. Densitometric analysis was performed and the phosphorylated proteins were normalized to their unphosphorylated counterparts. The densitometric intensity of p-38, p-42/44 and p-Thr308 did not significantly change under treatment with myrrh or IL-13 (data now shown). As shown in Figures 6A, B, IL-13 induced the STAT3 and STAT6 signaling of the pathway Janus kinase/signal transducer and activator of transcription (JAK/STAT). Compared to controls, the densitometric intensity of phosphorylated STAT3 in IL-13-treated cells increased (compared to control monolayers, n = 8–12, *p* = 0.0006). This increase was attenuated by preincubation with myrrh (compared to IL-13 exposure, n = 9–12, *p* = 0.0006) (Figure 6A). Secondly, an increased phosphorylation of STAT6 under IL-13 was observed (compared to controls, n = 8, *p* < 0.001). Treatment with myrrh could reduce also this increase although to a lesser extent (compared to IL-13 exposure, n = 8, *p* = 0.037) (Figure 6B).

Furthermore, an activation of the PI3K/Akt-pathway via phosphorylation of Ser473 was observed. IL-13-treated cells showed an increased signal for phosphorylated Ser473 (compared to controls, n = 12, *p* < 0.001), while preincubation with myrrh attenuated this increase (compared to IL-13 exposure, n = 12, *p* < 0.001) (Figure 6C).

### Myrrh attenuates IL-13-induced STAT phosphorylation and inhibits the IL-13-mediated claudin-2 upregulation

**FIGURE 7:**
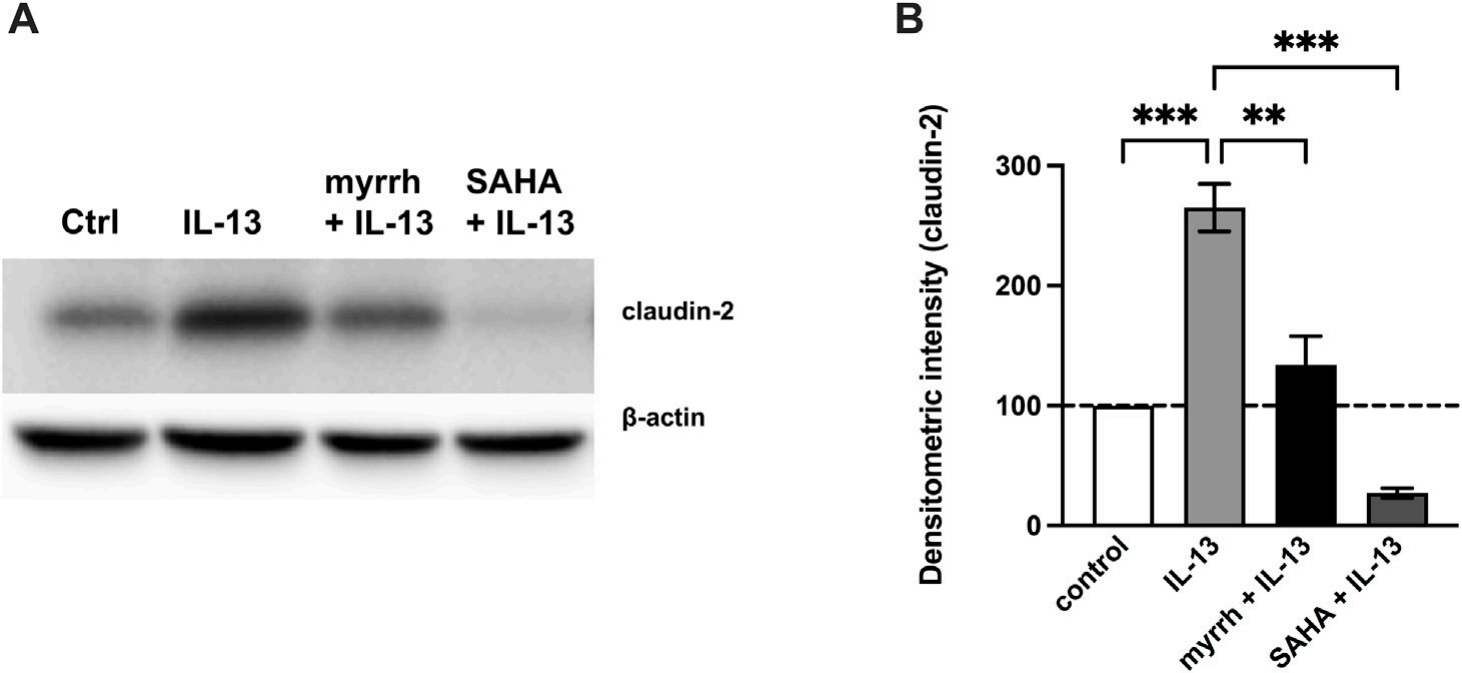
Inhibitory effect of STAT inhibition on claudin-2 expression **(A)** Representative Western blots of claudin-2 in HT-29/B6 cells after incubation with myrrh, IL-13 and SAHA. **(B)** Proteins were isolated and quantified using densitometric intensity analysis, normalized to β-actin. The IL-13-induced increase in claudin-2 expression is attenuated under treatment with myrrh and the STAT inhibitor SAHA. *n* = 12, **p < 0.01, ****p < 0.001 one-way ANOVA with Bonferroni correction.

Previous studies showed that the claudin-2 upregulation under inflammatory conditions is mediated by STAT signaling (Rosenthal et al., 2017; Krug et al., 2018). To further determine whether myrrh exerts its barrier protective effects by inhibiting the JAK/STAT-pathway, we inhibited STAT3 and STAT6 phosphorylation using the inhibitor suberoylanilide hydroxamic acid (SAHA, Vorinostat). Following the previously described protocol, HT-29/B6 cells were preincubated with SAHA or myrrh for 2 h and then challenged with 10 ng/mL IL-13. Subsequently, cells were lysed and Western blots of claudin-2 were performed. Myrrh preincubation reduced the claudin-2 expression compared to cells treated with IL-13 alone. However, cells incubated with SAHA and IL-13 did show a much stronger decrease in claudin-2 expression compared to cells treated with myrrh and IL-13, as shown in Figure 7. Therefore, together with the data from Figure 6, it can be hypothesized that myrrh attenuates the phosphorylation of STAT3 and STAT6 and consecutively reduces the IL-13-mediated claudin-2 upregulation.

## Discussion

### Herbal compound myrrh

Resinous extracts of herbal origin are an essential component in Ayurveda, traditional Chinese medicine, and Unani, the Perso-Arabic medicine (Batiha et al., 2023). The oleo-gum resin myrrh, known for its anti-inflammatory effects, has been used for treatment of gastrointestinal symptoms in traditional and modern medicine (al-Harbi et al., 1997).

Previous literature indicates the efficacy of myrrh against diarrhea in IBD, which requires further in-depth research to investigate the molecular processes behind the effects (Langhorst et al., 2013). In particular, the intestinal epithelial barrier and its involvement in inflammatory processes leading to leak-flux diarrhea are of interest. Recently, barrier-stabilizing effects of myrrh were demonstrated for intestinal epithelial cell monolayers (HT-29/B6, HT29-MTX-E12 and Caco-2 cell lines) (Rosenthal et al., 2017; Weber et al., 2020).

To further elucidate a potential protection of myrrh against cytokines present in UC, especially IL-13, our research aimed at diarrheal mechanisms in diseases such as UC. We could demonstrate for the first time that myrrh mitigates IL-13-induced barrier defects by protecting epithelial barrier function. This could be ascribed to two major effects: the reduction of epithelial apoptosis and the downregulation of the channel-forming TJ protein claudin-2.

A higher apoptotic rate, seen in cell monolayers incubated with IL-13, causes apoptotic leaks in the intestinal epithelial layer and thus may promote the influx of noxious agents such as toxins and antigens. In turn, this antigen influx leads to even higher inflammatory levels in the gut mucosa and can contribute to leak-flux diarrhea and the leaky gut phenomenon, which describes the vicious circle of inflammation-induced barrier dysfunction and the enhanced antigen influx through the weakened epithelial barrier (Bücker et al., 2014; Bücker et al., 2018). Thus, an anti-apoptotic effect of myrrh is of high importance for patients with active IBDs. Our experiments have shown a strong increase in apoptotic rate in IL-13-incubated cells compared to controls, an effect that was completely reversed by preincubation with myrrh.

As second main finding, the paracellular permeability for FD4 tends to be reduced presumably by the concomitant redistribution of tricellulin. Immunofluorescence stainings did show a shift of tricellulin from the tricellular TJ into the bicellular TJ under treatment with IL-13, while no significant change in expression levels was observed. These findings correspond to our and other experiments regarding the paracellular flux of the marker FD4 through the tricellular TJ (Awad et al., 2023). Tricellulin tightens the tricellular TJ against the influx of macromolecules and is regulated via the IL-13a2 receptor (Krug et al., 2018). As the redistribution of tricellulin is mitigated by myrrh, the protection against such macromolecule influx is ensured.

Besides tricellulin, the TJ protein claudin-2 is affected by IL-13. Claudin-2 forms a paracellular channel for small cations as well as for water, the upregulation of which causes barrier impairment in the colon by affecting the pore pathway (Amasheh et al., 2002; Rosenthal et al., 2010). It was shown that proinflammatory cytokines in IBD, such as TNFα, induce an increase of claudin-2 expression, promoting the leaky gut and the leak-flux type of diarrhea (Heller et al., 2005; Zeissig et al., 2007). In our HT-29/B6 cell monolayers, IL-13 induced a three-fold increase in claudin-2 expression. This increase was attenuated by myrrh preincubation to control levels. These results on IL-13-induced claudin-2-upregulation with subsequent increased barrier permeability here in our present study are paralleled by similar findings in an earlier study (Heller et al., 2005). However, as major finding of our present study, myrrh mitigates this upregulation and exerts a barrier-protective effect.

Furthermore, we investigated possible TJ-associated signaling pathways to determine how myrrh influences the alterations seen in TJ protein expression. IL-13 interferes with the STAT pathway, mainly induces phosphorylation of STAT3 and STAT6. Treatment with myrrh is effective in blocking STAT-phosphorylation under challenge with IL-13. Moreover, the SAHA-mediated STAT-inhibition prevents the upregulation of claudin-2 caused by IL-13 as well. Thus, it is suggestive that myrrh exerts its protective effects against IL-13 and its observed TJ expression alterations by inhibiting STAT phosphorylation.

STAT3 is phosphorylated by members of the IL-6 family and is mainly known for the promotion of immunosuppression and its effect in colorectal cancer progression following constitutive activation (Zou et al., 2020). Especially relevant in carcinogenesis and chemoresistance, in both CD and UC patients the activation of STAT3 leads to prolonged survival of pathogenic T cells (Atreya et al., 2000; Musso et al., 2005). Furthermore, in asthma and allergic inflammation, a higher STAT3 activation in Th2 cells was induced under treatment with IL-13 (León and Ballesteros-Tato, 2021). Our study showed the STAT3 activation in intestinal epithelial cells, caused by IL-13 and inhibition by myrrh.

The phosphorylation and activation of STAT6 is mainly induced by IL-13 and IL-4 (Karpathiou et al., 2021). A murine model of UC showed the STAT6-dependent induction of claudin-2 expression (Rosen et al., 2013). Additionally, in UC, IL-13 and TNFα express synergistic effects on activation of STAT6, causing altered TJ protein expression, especially of claudin-2 (Domazetovic et al., 2020). While protective and barrier-stabilizing effects of myrrh against the cytokine TNFα have previously been shown (Rosenthal et al., 2017), our present study extends these findings to IL-13, a main mediator in UC. As TNFα is correspondingly increased in patients with IBDs, the importance of myrrh as a potential additional treatment option in IBD is highlighted with the present findings.

### Other plant-derived remedies with an effect on STAT6

Myrrh is only one of several compounds currently being investigated for their effects on the epithelial barrier function and structure in different phases of IBD. Herbal remedies are of particular interest in IBD research, mainly because they impose fewer side effects.

Curcumin, an herbal compound obtained from turmeric, was shown to influence epithelial barrier and TJ proteins. Against TNFα and *C. jejuni*, curcumin had a barrier-stabilizing effect via up-regulating TJ proteins claudin-4 and -8 as well as occludin and ZO-1 through NF-κB signaling (Lobo de Sá et al., 2019; Iglesias et al., 2022). Furthermore, it was observed to restore immunologic balance in experimental colitis by modulating JAK/STAT/SOCS signaling (Zhao et al., 2016).

With a similar mechanism of action to myrrh, Vitamin D (1,25(OH)_2_D_3_) can modulate claudin expression in patients with UC (Stio et al., 2016). Vitamin D effects the STAT6 and Smad7 pathways (Domazetovic et al., 2020). Especially the vitamin D-induced inhibition of STAT6 was linked to the claudin-2 downregulation (Domazetovic et al., 2020). This reiterates the potential of STAT6 inhibition as a target structure in IBD treatment.

## Conclusion

In conclusion, we showed the barrier-protective effects of myrrh against the cytokine IL-13, a major effector cytokine in UC, which causes claudin-2 upregulation and apoptosis induction. Incubation with myrrh results in a downregulation of claudin-2 and a reduced apoptotic rate, regulated via the STAT3/STAT6 pathways. With its limited side effects compared to immunosuppressive medication, the herbal compound could be a valuable addition to the treatment of IBD patients and diarrhea.

## Data Availability

The raw data supporting the conclusion of this article will be made available by the authors, without undue reservation.
